# Live Cell Imaging Reveals HBV Capsid Translocation from the Nucleus To the Cytoplasm Enabled by Cell Division

**DOI:** 10.1128/mbio.03303-22

**Published:** 2023-02-21

**Authors:** Sofia Romero, Nuruddin Unchwaniwala, Edward L. Evans, Kevin W. Eliceiri, Daniel D. Loeb, Nathan M. Sherer

**Affiliations:** a McArdle Laboratory for Cancer Research (Department of Oncology), University of Wisconsin-Madison School of Medicine and Public Health, Madison, Wisconsin, USA; b Institute for Molecular Virology, University of Wisconsin-Madison, Madison, Wisconsin, USA; c Microbiology Doctoral Training Program, University of Wisconsin-Madison, Madison, Wisconsin, USA; d Laboratory for Optical and Computational Instrumentation, Center for Quantitative Cell Imaging, University of Wisconsin-Madison, Madison, Wisconsin, USA; e Morgridge Institute for Research, Madison, Wisconsin, USA; f Carbone Cancer Center, University of Wisconsin-Madison School of Medicine and Public Health, Madison, Wisconsin, USA; University of Pittsburgh; University of Pennsylvania

**Keywords:** core protein, hepatitis B virus, live cell imaging, nuclear export, subcellular trafficking, virus assembly

## Abstract

Hepatitis B virus (HBV) capsid assembly is traditionally thought to occur predominantly in the cytoplasm, where the virus gains access to the virion egress pathway. To better define sites of HBV capsid assembly, we carried out single cell imaging of HBV Core protein (Cp) subcellular trafficking over time under conditions supporting genome packaging and reverse transcription in Huh7 hepatocellular carcinoma cells. Time-course analyses including live cell imaging of fluorescently tagged Cp derivatives showed Cp to accumulate in the nucleus at early time points (~24 h), followed by a marked re-distribution to the cytoplasm at 48 to 72 h. Nucleus-associated Cp was confirmed to be capsid and/or high-order assemblages using a novel dual label immunofluorescence strategy. Nuclear-to-cytoplasmic re-localization of Cp occurred predominantly during nuclear envelope breakdown in conjunction with cell division, followed by strong cytoplasmic retention of Cp. Blocking cell division resulted in strong nuclear entrapment of high-order assemblages. A Cp mutant, Cp-V124W, predicted to exhibit enhanced assembly kinetics, also first trafficked to the nucleus to accumulate at nucleoli, consistent with the hypothesis that Cp’s transit to the nucleus is a strong and constitutive process. Taken together, these results provide support for the nucleus as an early-stage site of HBV capsid assembly, and provide the first dynamic evidence of cytoplasmic retention after cell division as a mechanism underpinning capsid nucleus-to-cytoplasm relocalization.

## INTRODUCTION

Hepatitis B virus (HBV) is a global public health concern that significantly increases the risk for developing cirrhosis and hepatocellular carcinoma among chronically infected people worldwide, leading to approximately one million deaths annually – indicated by a 2019 report by the World Health Organization (https://www.who.int/news-room/fact-sheets/detail/hepatitis-b). While a vaccine offers protection against infection, it is not therapeutic. Thus, determining novel HBV-host interactions that support infection may expose potential targets relevant to the design of new antiviral strategies.

HBV is an enveloped, reverse-transcribing DNA virus belonging to the *Hepadnaviridae* family. Upon infection, HBV nucleocapsids deliver relaxed-circular DNA (rcDNA) genome intermediates to the nucleus to initiate replication (1, 2). After nuclear entry, rcDNA is converted to covalently closed circular (ccc) DNA, and associates with cellular histone proteins to serve as the template for viral RNA transcription (3–6). In addition to sub-genomic mRNAs and Pre-Core RNA, HBV synthesizes pre-genomic RNA (pgRNA) that serves as the viral mRNA encoding Core protein (Cp) and Polymerase (P) protein, and also represents the RNA genome intermediate successively packaged by Cp and P into nascent nucleocapsids (1, 2, 7). However, it is important to note that a significant proportion of HBV capsids are devoid of viral genome, with up to 90% of cell-associated capsids empty when studied *in vitro* or *in vivo* (8, 9). After genomic maturation (reverse transcription of pgRNA by P to generate rcDNA) (10), nucleocapsids are either enveloped at host-derived membranes and studded with viral envelope proteins – Pre-S1 (Large), Pre-S2 (Medium), and S (Small) – or retargeted to the nucleus for cccDNA enrichment – a process known as intracellular amplification (11–14).

**FIG 1:**
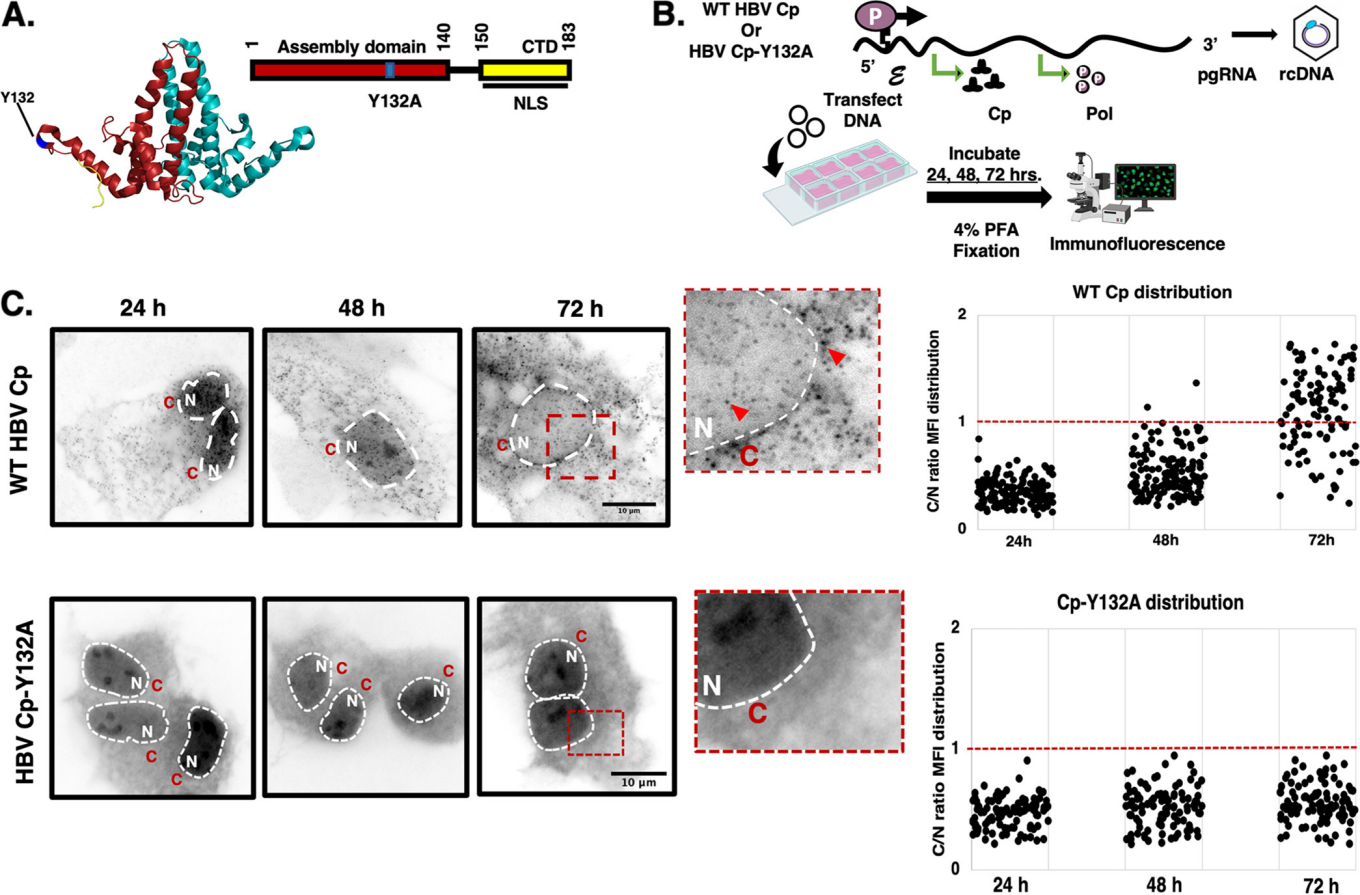
Changes to the subcellular distribution of HBV Cp over time. (A) Cartoon illustration of Cp divided into the N-terminal Assembly Domain and C-Terminal Domain (CTD). The CTD encodes the nuclear localization signal (NLS). The HBV Cp dimer crystal structure shown is based on PBD:3J2V. The position of the Cp-Y132A mutation is highlighted in blue, shown on the red Cp subunit. (B) Outline of HBV expression and time course analysis workflow based on immunofluorescence (IF) detection of WT HBV Cp or Cp-Y132A. Images were developed using BioRender. (C) Representative images and quantification from IF time-course analysis of Huh7 cells expressing WT Cp with packageable pgRNAs, or the Cp-Y132 (no assembly) control. Cells were fixed at the indicated time points with WT Cp or Cp-Y132A detected using polyclonal anti-HBc antiserum. White dashed lines differentiate nuclei (N) from cytoplasm (C). Red dashed boxes highlight regions of interest, with red arrows indicating WT Cp puncta consistent with assembled capsids. Image scale bars represent 10 μm. Plots on the right present ratios of C/N mean fluorescence intensity (MFI) for 100 cells per condition per time point. The red dashed line at 1 indicates equivalent levels of nuclear and cytoplasmic fluorescence signals. Greater than 1 indicates more cytoplasmic MFI relative to the nucleus. Less than 1 indicates more nuclear MFI relative to the cytoplasm.

The HBV capsid represents an icosahedron with a dominant species measuring approximately 32 nm in diameter comprised of 120 core protein (Cp) dimers, and a minor population of capsid particles measuring 30 nm and consisting of 90 Cp dimers (15). The Cp subunit is 21-kDa, and consists of 183 amino acids split into 2 functional domains: the N-terminal (assembly) domain (1-140 aa) that mediates multimerization of Cp dimers into capsids and the C-terminal domain (CTD) that facilitates pgRNA packaging, reverse transcription, and encodes the nuclear localization signal (NLS) that targets HBV nucleocapsids to the nucleus to initiate infection (16–18) (Fig. 1A).

Prior to, and during, the assembly of nascent virus particles, the subcellular trafficking dynamics of Cp are only partially understood. HBV capsid assembly has traditionally been thought to occur principally in the cytoplasm, considering that the cytosol is where nucleocapsids are enveloped during virion egress. However, Cp has been, in several studies, also been shown to accumulate to high levels in the nucleus of hepatocytes both in cell culture and *in vivo* (19–23). A recent study by Nair and Zlotnick (24) showed that Cp’s abundance in the cytoplasm is a function of time and dependent on Exportin-1 (XPO1, also known as CRM1), a host nuclear export receptor; while work from the Shih lab has implicated both CRM1 and the cellular NXF1-p15 nuclear export machinery in Cp nuclear export and virion secretion (25–27). Nucleus-associated Cp, potentially capsids, have also been observed to shift to the cytoplasm in mitotically active hepatocytes, suggesting that the cell cycle is also a determinant of Cp subcellular localization (28, 29).

In this study, our objective was to better define HBV assembly dynamics using a combination of single cell fixed and live imaging techniques. Using Huh7 hepatocellular carcinoma cells as a model system, we demonstrate that assembly-competent Cp is localized primarily to the nucleus soon after its synthesis, where Cp can form high-order Cp assemblages consistent with capsids. Capsid distributions shift to the cytoplasm at later time points, mediated through nuclear escape coincident with nuclear envelope breakdown during cell division, and followed by strong cytoplasmic retention. Blocking cell proliferation using aphidicolin (APC), a drug that arrests cells in S phase prior to nuclear membrane breakdown led to entrapment of Cp in the nucleus consistent with cell division representing a major means by which nuclear HBV capsids/high-order assemblages can be delivered to the cytoplasm should liver cells be mitotically active. These results reveal a first glimpse of real-time Cp transport dynamics, and reemphasize the potential implications for the cell cycle in HBV replication (6, 30, 31).

## RESULTS

### HBV Cp accumulates preferentially in the nucleus at early time points post-expression.

The productive phase of the HBV viral life cycle is initiated in the nucleus by host-mediated transcription of pgRNA that serves as both the mRNA encoding Cp and P, as well as the RNA genome substrate packaged into assembling capsids. To better define subcellular sites of HBV assembly, our first objective was to carry out single cell time course analyses of HBV Cp localization over time, comparing wild-type (WT) Cp trafficking to that of a well characterized Cp mutant (Cp-Y132A) rendered assembly-incompetent, due to removal of the phenolic side chain provided by tyrosine-132 needed by Cp to form interdimer interactions (Fig. 1A) (32).

To this end, we first transfected Huh7 liver carcinoma cells to express either the LJ144 HBV genetic clone encoding wild-type assembly-competent Cp (hereafter referred to as “WT Cp”) or the J305 clone encoding Cp-Y132A co-expressed with LJ145 that encodes a Cp-minus packageable genome substrate (17, 33). LJ144 expresses Cp and P from pgRNAs competent for packaging and reverse transcription, but lacks viral glycoprotein expression, validated in prior work to serve as a reliable model for studying nucleocapsid formation and viral genome replication (rcDNA synthesis) (16, 34). Cells were fixed at 24, 48, or 72 h post-expression, and processed for indirect immunofluorescence (IF), detecting Cp using an anti-HBc polyclonal antibody that detects Cp’s c/e1 immunodominant epitope (35) (Fig. 1B). Huh7 cells were chosen for these experiments because they have been used extensively to study HBV, and work well for both fixed and live cell imaging, being relatively flat cells that are stationary when plated on glass. Interestingly, we observed WT Cp to be predominantly localized to the nucleus at the earlier (24 and 48 h) time points, only accumulating to relatively high levels in the cytoplasm at 72 h (Fig. 1C, *n *= 50). In contrast, the Cp-Y132A mutant was predominantly detected in the nucleus independent of time. Consistent with capsid assembly events, WT Cp formed distinct puncta in both nuclear and cytoplasmic compartments (Fig. 1C, top panel), but with no puncta formation observed for Cp-Y132A (Fig. 1C, bottom panel). Interestingly, both nuclear WT Cp and Cp-Y132A were enriched in association with subnuclear granules (most predominantly in early periods of gene expression (Fig. 1C, 24h and 48h time points) resembling nucleoli. These results demonstrated that Cp assembly-competency is a key determinant of Cp subcellular distribution, and suggested an early bias to accumulating preferentially in the nucleus.

### Cp forms high-order structures consistent with capsids in the nucleus.

**FIG 2:**
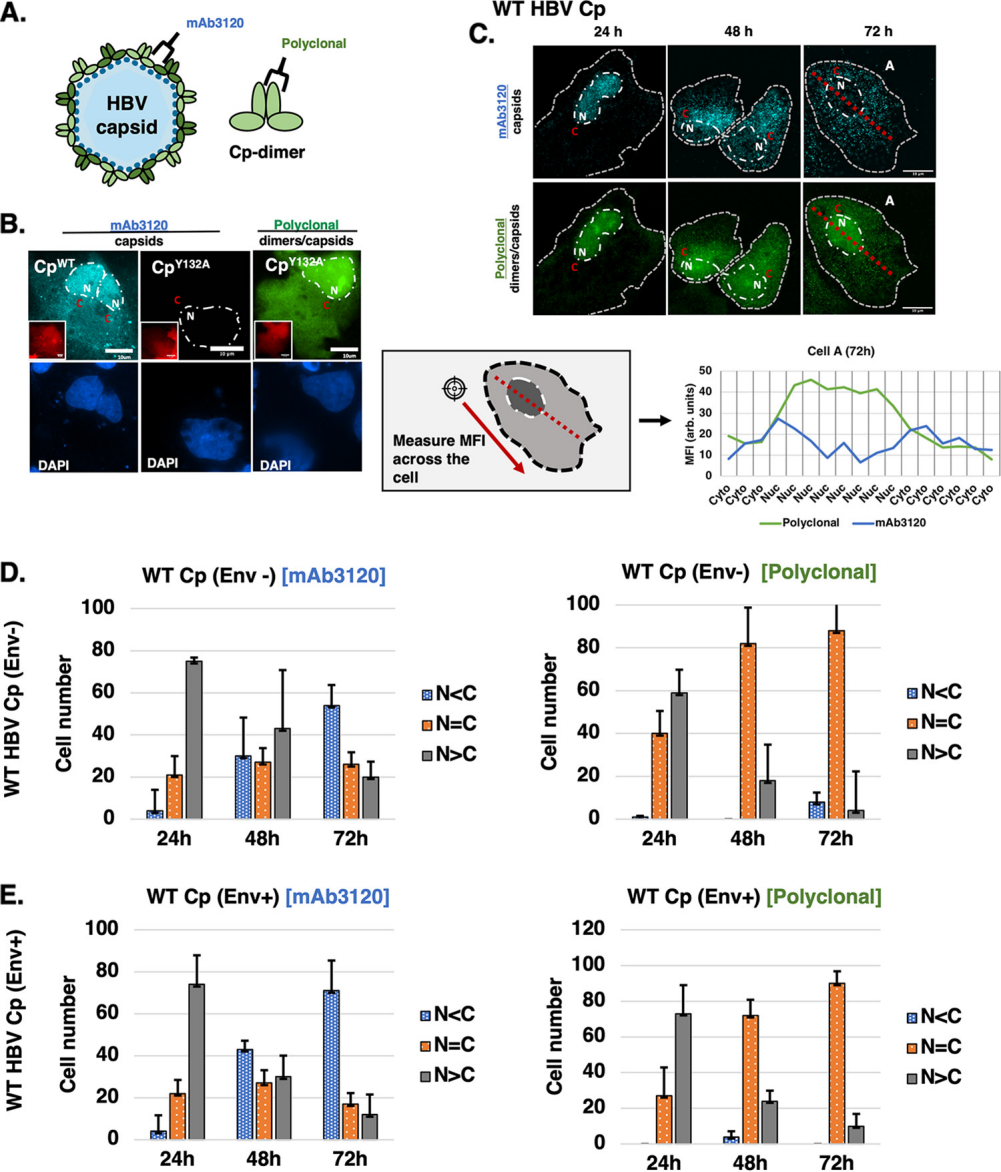
Cp forms high-order assemblages in the nucleus. (A) Illustrations of Cp dimer and capsid structures highlighting the HBV Cp binding sites for the mAb3120 and polyclonal (anti-HBc) antibodies used for the dual labeling strategy. (B) Representative images from IF analysis of cells expressing WT Cp or Cp-Y132A, and incubated with the indicated antibodies, to confirm binding specificity. mCherry (inset, red) was co-transfected with the Cp variant to assist in identifying transfected cells prior to fixation, staining with DAPI (blue), and staining for IF (green). mAb3120 was confirmed as unable to detect Cp-Y132A (central panels). (C) Images and analysis of dually labeled Huh7 cells expressing WT Cp and detected using mAb3120 (cyan) and polyclonal anti-HBc (green). Transect analysis illustrates differential detection of Cp with polyclonal (predominantly nuclear) and mAb3120 (predominantly cytoplasmic) at 72h in a representative cell; consistent with unassembled Cp trafficking to the nucleus, even when the assembled capsid population is predominantly in the cytoplasm. Scale bars represent 10 μm. (D) Bar graphs quantifying the subcellular localization of WT Cp for 100 cells per condition, detected using the indicated antibody and corresponding to the experiment in (C). Error bars represent the standard deviation of the mean for 3 biological replicates. (E) Bar graphs as for (D) confirming a similar subcellular localization of WT Cp distribution over time when expressed from a plasmid encoding envelope glycoproteins (Env+).

Because assembly-competent Cp formed puncta in the nucleus at the earliest time points, we next sought to determine if nuclear Cp was forming high-order structures consistent with capsids. To this end, we developed a sequential co-labeling IF assay, wherein we first stained with a capsid-specific monoclonal antibody (mAb3120) prior to staining with the anti-HBc polyclonal antibody (Fig. 1) that was unable to distinguish between capsids or Cp monomers/dimers (with differential targets depicted in Fig. 2A). The selectivity of mAb3120 is due to its binding to a discontinuous epitope that straddles the interface between 2 adjacent Cp dimer units (36–38). We confirmed this specificity by demonstrating that mAb3120 detected WT Cp under assembly-competent conditions but not the assembly-defective Cp-Y132A mutant protein (Fig. 2B).

Sequential co-labeling of WT Cp-expressing Huh7 cells over our 72 h time course revealed similar levels of WT Cp detected in the nucleus by both antibodies at the 24 h posttransfection time point, suggesting that the nucleus represents a site competent for formation of capsids and/or alternative high-order assemblages. Similar to our initial analysis using the polyclonal antibody in Fig. 1, we also observed a clear shift in the mAb3120 signal from the nucleus to cytoplasm over time (65% N ≤ C by 48 h, and 92%N ≤ C by 72 h) (Fig. 2C and D), consistent with relocalization of capsids formed in the nucleus being relocated to the cytoplasm. Unassembled Cp, detected by the polyclonal but not mAb3210, was more likely to be detected in the nucleus at all time points, suggesting that Cp monomers/dimers are constitutively trafficked to the nucleus. Because our initial conditions lacked viral glycoprotein expression, we also evaluated Cp distribution using our dual antibody approach for cells expressing TMA153 – a LJ144 equivalent viral clone that also expresses HBV envelope proteins. Sequential IF detection of Cp/high-order assemblages using mAb3120 and anti-HBc polyclonal antibodies demonstrated similar nuclear-to-cytoplasm trafficking pattern of Cp/capsids (70% N ≤ C by 48 h, and 88%N ≤ C by 72 h) (Fig. 2E), compared to minus envelope expression conditions. These data further indicated that it is the high-order assemblage of capsid components that underpins the major transition from the nucleus to the cytoplasm over time.

**FIG 3:**
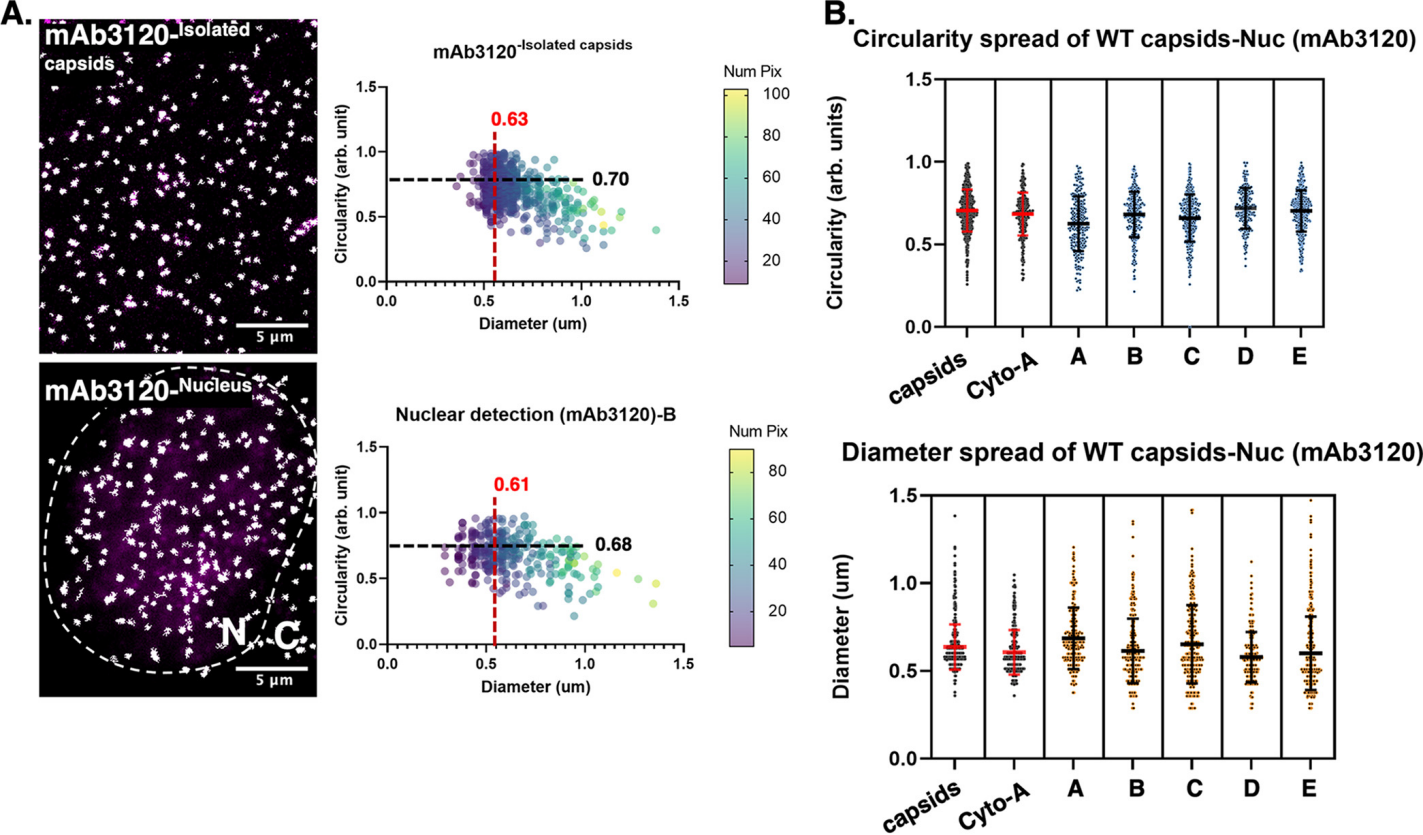
Intracellular nuclear puncta exhibit similar morphology to HBV capsids isolated by velocity-sedimentation. (A) IF detection of WT Cp using mAb3120 expressed in Huh7 cells for either isolated capsids from velocity sedimentation (fraction #7), or within the nuclear compartment of Cp-expressing cells fixed at 24 h posttransfection. Images were background subtracted and subjected to image analysis comparing the circulatory and diameter of objects (puncta), as described in Materials and Methods. Mean values are denoted by black and red dashed lines, respectively. A circularity value of 1.0 represents a perfect circle. (B) Circularity and diameter distributions for >200 puncta from 5 independent nuclei presented in cyan and dark orange dot plots, respectively, compared to puncta in the cytoplasm (Cyto-A) or isolated capsids (capsids). Error bars represent the standard deviation of the mean.

To provide confidence that nucleus-associated WT Cp assemblages were indeed capsids, we compared the size and circularity of Cp nuclear signals in cells 24 h posttransfection to those of isolated, gradient-purified HBV capsids (Fig. 3). Cells expressing WT Cp and isolated capsids were subjected to dual antibody IF, and background subtracted prior to bright objects being subjected to multivariate image analysis to determine shape (circularity) and size (pixel number and maximum diameter) (Fig. 3A). As expected, isolated capsids detected using mAb3120 yielded circular puncta of uniform size that were consistent in size and shape with cell-associated WT Cp structures identified in either the nucleus or the cytoplasm (Fig. 3A, right panels, and Fig. 3B). It is important to note, however, that these assays were unable to differentiate between nuclear capsids carrying packaged and reverse transcribed pgRNA, representing replication-competent nucleocapsids, or empty capsids.

### Delivery of nuclear HBV capsids to the cytoplasm occurs predominantly during nuclear membrane breakdown in conjunction with cell division.

Having obtained evidence that capsids assemble in the nucleus prior to the bulk of the signal being relocalized to the cytoplasm in a time-dependent manner (over 72 h), we considered 3 competing hypotheses that might explain the nucleus-to-cytoplasm transition: (i) that nuclear capsids/high-order assemblages formed early were degraded at later time points relative to the cytoplasmic population; (ii) that capsids/high-order assemblages formed in the nucleus transitioned either gradually or *en masse* from the nucleus to the cytoplasm through nuclear pore complexes (NPCs) as recently proposed (19–21); or (iii) that capsids/high-order assemblages left the nucleus during nuclear membrane dissolution as a consequence of cell division, consistent with prior reports from Yeh, C-T et al. and Guidotti, LG et al. (19, 39).

**FIG 4:**
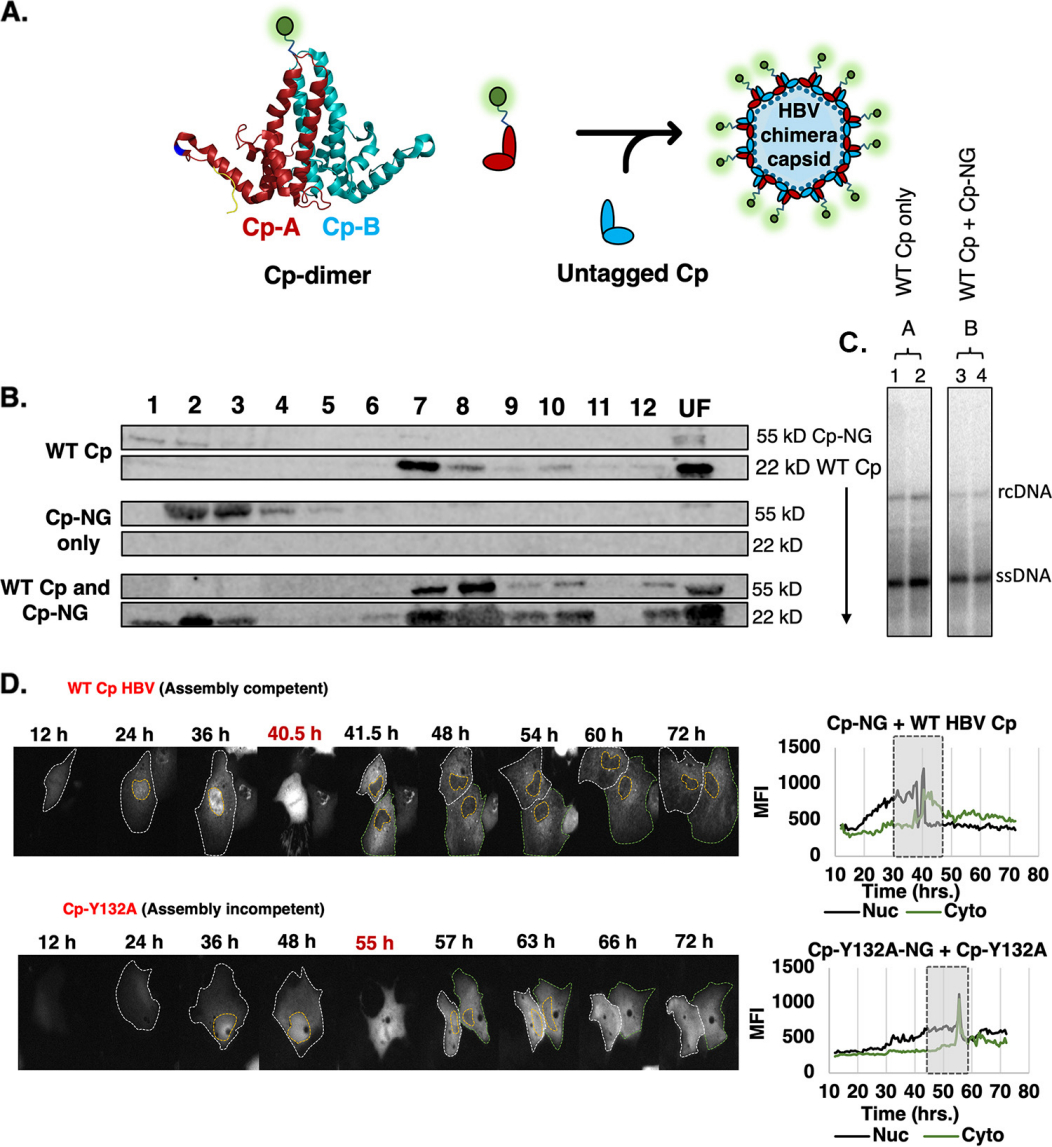
Live cell analysis reveals that assembly-dependent relocalization of Cp to the cytoplasm occurs predominantly during cell division. (A) Illustration detailing the co-expression of engineered Cp-NG (NeonGreen) and untagged Cp to support assembly of chimeric fluorescent HBV capsids. The NG tag is represented as a green circle attached to the Cp apical region between Cp amino acids D78 and P79. (B) Velocity sedimentation analysis of Cp multimerization, showing that tagged HBV (Cp-NG, ~55 kD), when expressed alone, sediments in fractions 2 to 4, but shifts to capsid fractions 7 and 8 when co-expressed with untagged Cp (~22 kD), consistent with formation of fluorescent chimeric capsids. (C) Southern blot detection of HBV reverse transcribed viral DNA for (A) WT Cp only or (B) WT Cp + Cp-NG (chimeric capsid) conditions. Bands corresponding to rcDNA or ssDNA are indicated. (D) Images from live cell detection and tracking of assembly-competent Cp-NG (top panel) or assembly-incompetent Cp-Y132A-NG (lower panel). Time-course images correspond to Movies S1 and S2. Time points labeled in red designate the initiation of a cell division event. On the right, MFI for nuclear (black line) or cytoplasmic (green line) Cp-NG or Cp-Y132A-NG signals are plotted for a representative cell tracked over 72 h of live cell imaging. Gray box highlights a 10 h window that encompasses a cell division event.

Because our understanding of Cp transport had been limited to fixed cell approaches that are unable to provide resolution of live cellular dynamics, we sought to engineer a reliable long-term (>24 h) live cell HBV imaging strategy based on fluorescently labeled Cp derivatives, again with the goal of comparing changes to the subcellular distribution of assembly-competent WT Cp to that of the assembly-incompetent Cp-Y132A control. A study from Nassal and colleagues had demonstrated previously that insertion of a 238 aa. green fluorescent protein (GFP) into the tip of the prominent Cp dimer surface spike (found between aa. 78 and 79 of Cp) can be tolerated by HBV capsids when co-expressed with untagged Cp to form Cp/Cp-GFP chimeric fluorescent particles (40). Informed by this insight, we generated analogous WT and Cp-Y132A variants carrying NeonGreen (Cp-NG and Cp-Y132A-NG) (Fig. 4A), choosing NG over GFP because it is a brighter fluorophore that also has better protein folding kinetics (41). Validation experiments confirmed that Cp-NG and unlabeled Cp, co-expressed at a 1:1 ratio, yielded Cp assemblages that co-sedimented (at fractions 7 and 8) consistent with Cp-NG being incorporated successfully into chimeric capsids (Fig. 4B). Additionally, Southern blot detection of newly synthesized HBV DNA confirmed that co-expression of Cp-NG and untagged Cp had little to no impact on HBV DNA synthesis when compared to the DNA synthesis supported by untagged WT Cp expression (positive control) (Fig. 4C). These data confirmed that co-expression of Cp-NG and untagged Cp yielded a system that both formed chimeric capsids and supported HBV genome replication, but did not allow us to determine the percentage of Cp-NG chimeric capsids competent for genome packaging and reverse transcription.

10.1128/mbio.03303-22.1 MOVIE S1 Representative video corresponding to Fig. 3 showing live cell imaging of WT Cp-NG expression in a Huh7 cell, with images acquired every 30 min over 72 h of continual imaging. Arrows and outlines highlight a cell division event. Download Movie S1, AVI file, 1.3 MB. Copyright © 2023 Romero et al. 2023 Romero et al. https://creativecommons.org/licenses/by/4.0/ This content is distributed under the terms of the Creative Commons Attribution 4.0 International license.

10.1128/mbio.03303-22.2 MOVIE S2 Representative video corresponding to Fig. 3 showing live cell imaging of WT Cp-Y132A-NG expression in a Huh7 cell, with images acquired every 30 min over 73.5 h of continual imaging. Arrows and outlines highlight a cell division event. Download Movie S2, AVI file, 1.9 MB. Copyright © 2023 Romero et al. 2023 Romero et al. https://creativecommons.org/licenses/by/4.0/ This content is distributed under the terms of the Creative Commons Attribution 4.0 International license.

We next proceeded with live cell imaging of single Huh7 cells expressing either Cp-NG or Cp-Y132A-NG, monitored for up to 70 h post-expression. Consistent with our IF experiments (Fig. 1 and 2), WT Cp-NG was initially observed to accumulate in the nucleus at earlier time points (up to 36 h), prior to shifting to the predominantly cytoplasmic distribution at a later time point; unlike the Cp-Y132A-NG control that did not exhibit major differences to nuclear or cytoplasmic abundances (Fig. 4D). Strikingly, our video microscopy also revealed that WT Cp-NG’s re-localization to cytoplasm was not a gradual process, as would be expected if capsids were degraded in the nucleus over time or being translocated to the cytoplasm through nuclear pores (i.e., hypotheses i or ii), but instead was observed predominantly in conjunction with nuclear membrane breakdown during cell division (most consistent with hypothesis iii) (Fig. 4D, upper panel, and Movie S1). Moreover, after nuclear membrane reformation, the majority of Cp-NG remained in the cytoplasm suggestive of a capsid retention mechanism, with Cp-NG only gradually beginning to build up in the nucleus again over time (Fig. 4D, upper panel). In contrast, the Cp-Y132A-NG mutant immediately was observed to re-accumulate in the nucleus after cell division (Fig. 4D, lower panel, and Movie S2). These videos demonstrated that, under these conditions, nucleocytoplasmic re-distribution of Cp/capsids was principally facilitated by nuclear membrane breakdown during cell division, and indicated that Cp/capsids were preferentially retained in the cytoplasm after cell division in an assembly-dependent manner.

### Blocking cell proliferation with aphidicolin results in nuclear entrapment of HBV capsids.

**FIG 5:**
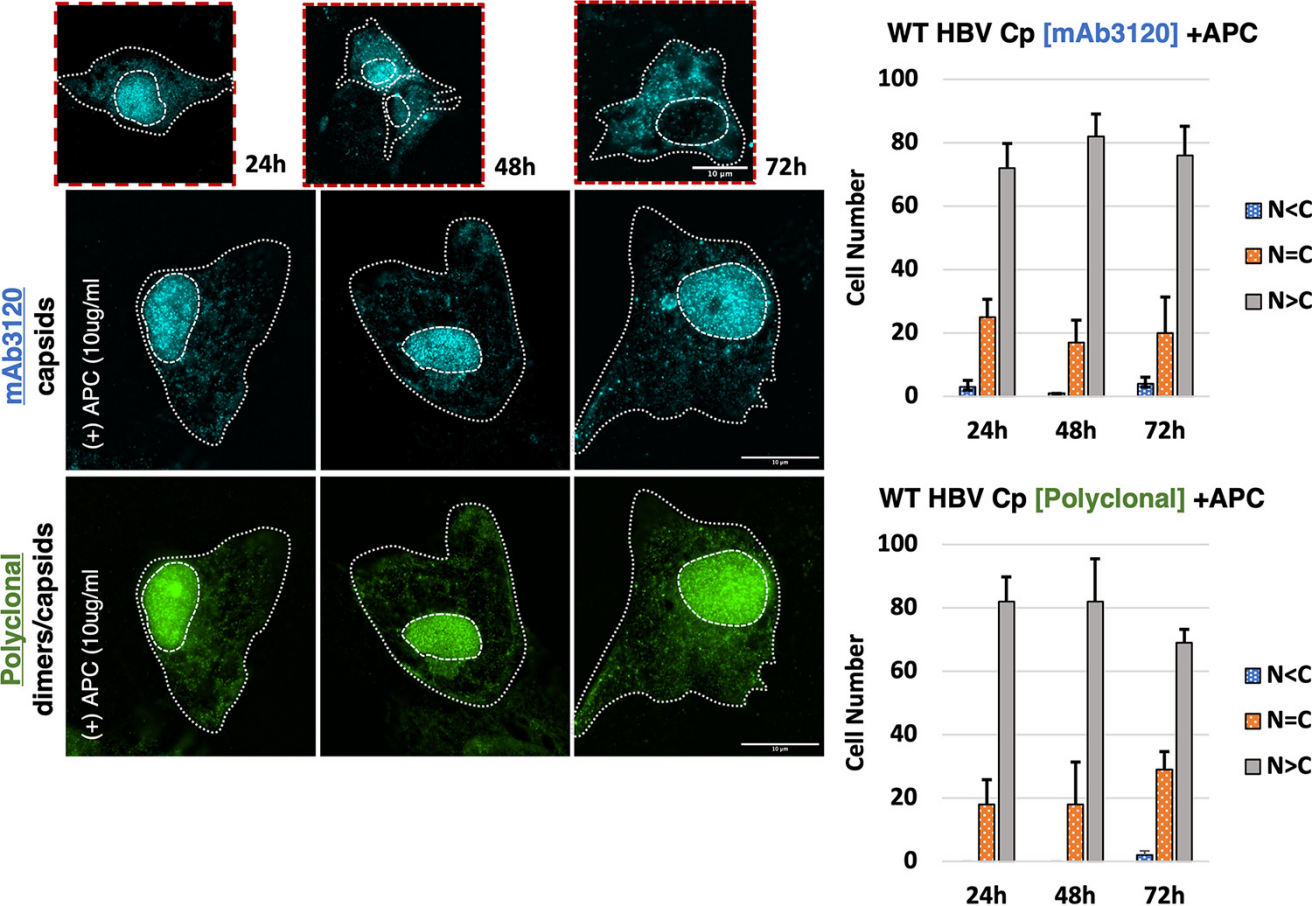
Inducing cell cycle arrest using aphidicolin that entraps HBV Cp/capsids in the nucleus. Representative images showing Huh7 cells expressing WT HBV, treated with 10 μg/mL APC for the indicated time period (e.g., 24, 48, and 72 h post-gene expression). In the presence of APC, WT Cp remained predominantly nuclear at all time points, as detected by both capsid-specific mAb3120 and anti-HBc polyclonal antibody. Smaller images (top) bordered in red show WT HBV control WT Cp expression and relocalization from the nucleus to the cytoplasm in the absence of APC. Scale bars represent 10 μm. Bar graphs present data from 3 independent experiments, measuring 100 cells per condition and time point, with error bars representing the standard deviation of the mean.

We hypothesized that if nuclear envelope breakdown was capsid’s predominant route of nuclear escape, then nuclear-to-cytoplasmic re-distribution would be blocked when cell proliferation was arrested. To test this hypothesis, we chose to inhibit cell division using aphidicolin (APC) – an inhibitor of host DNA polymerase activity, that blocks the cell cycle in early S phase and prior to nuclear membrane breakdown (42, 43). WT Cp was expressed in the absence or presence of 10 μg/mL APC by dual label IF over a 72 h time course, identical to the approach described for Fig. 2 (Fig. 5). In comparison to normal cell proliferation conditions, wherein capsids/high-order assemblages were re-localized from the nucleus to the cytoplasm, cells treated with 10 μg/mL APC exhibited a marked retention of capsids in the nucleus (>70% N > C) at all time points (Fig. 5). These results were consistent with a model wherein HBV capsids formed in the nucleus at early time points require cell division to achieve redistribution to the cytoplasm.

### A Cp mutant (V124W) that exhibits rapid assembly kinetics also accumulates in the nucleus at early time points.

**FIG 6:**
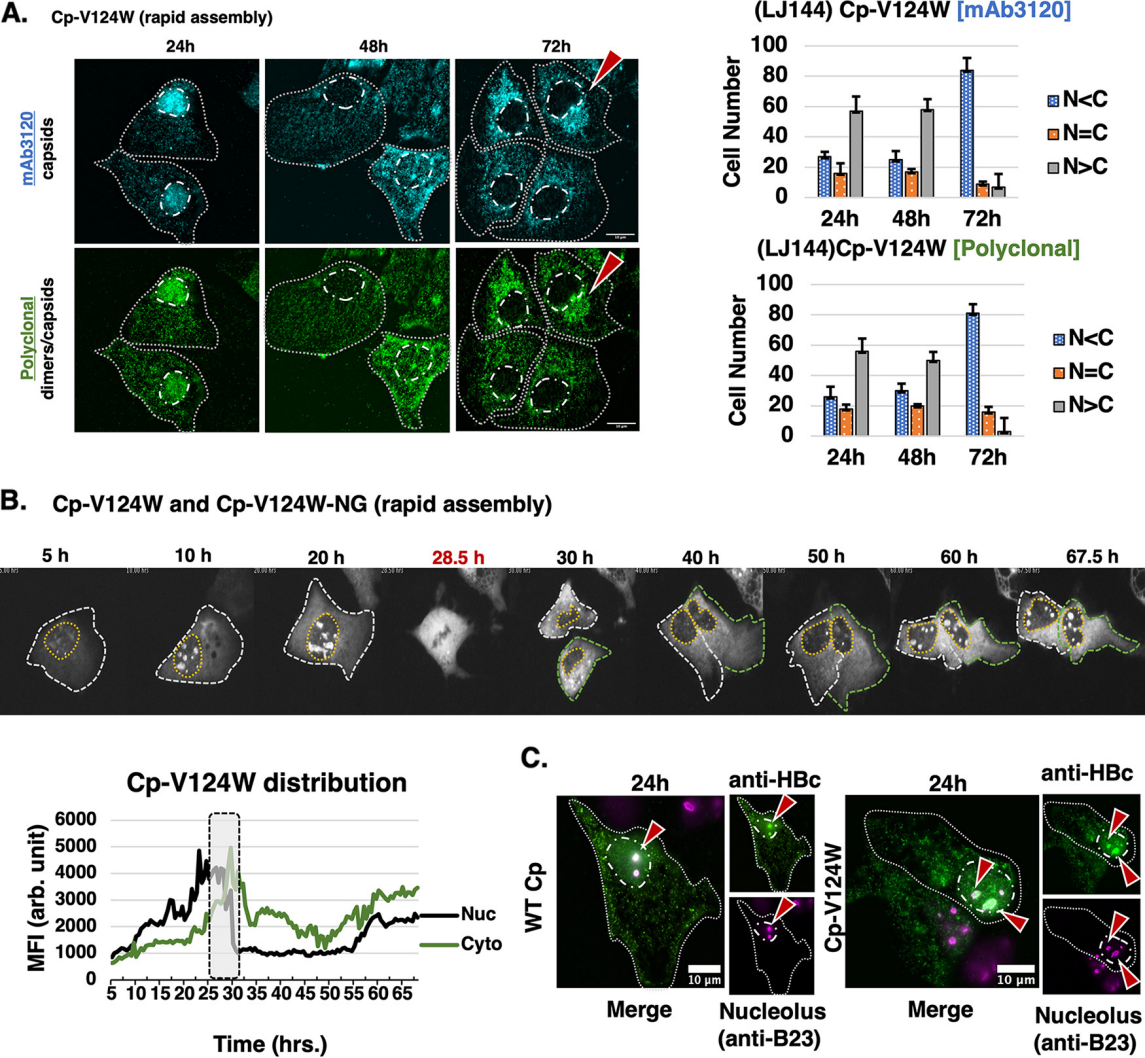
Increasing rates of capsid assembly does not affect Cp’s preferential accumulation in the nucleus at early time points. (A) Representative images of cells expressing rapid assembly mutant Cp-V124W tracked over a 72 h time course using dual label IF analysis. The red arrows indicate cytoplasmic co-localization of capsid/Cp detected by both mAb3120 and polyclonal anti-HBc antisera, respectively. Graphs present relative levels of nuclear versus cytoplasmic Cp-V124W distribution for 100 cells per time point. Error bars represent the standard deviation of the mean for 3 independent experiments. (B) Images from live single cell detection of fluorescent Cp-V124W-NG co-expressed with untagged Cp-V124W over an ~70 h time course. Time point labeled in red (28.5h) designates a cell division event. Please reference Movie S3 for the complete video file. Graph shows tracking of nuclear and cytoplasmic Cp-V124W-NG MFI for a representative cell, plotted over time. Gray box highlights an ~6 h time window encompassing a cell division event. (C) Images from IF detection of untagged WT Cp and Cp-V124W using polyclonal anti-HBc (green) with cellular nucleoli labeled using anti-Nucleophosmin (B23) (magenta) antibodies, indicating that both WT Cp and Cp-V124W localize to the nucleolus at early time points. Scale bars represent 10 μm. Red arrows highlight co-incident detection of Cp or Cp-V124W with Nucleophosmin (B23).

Because we observed that WT capsids/high-order assemblages were predominantly retained in the cytoplasm after cell division (Fig. 2C), we hypothesized that increasing rates of HBV capsid assembly would bias Cp to accumulate in the cytoplasm early (i.e., prior to cell division) should assembly rates outpace the rate of Cp monomer/dimer nuclear import. To test this hypothesis, we tracked a Cp mutant, Cp-V124W that carries a point-mutation at amino acid 124 within the interdimer region of Cp that kinetically favors protein-protein interactions, and has been shown to increase rates of capsid assembly *in vitro*. (44) Sequential IF staining of untagged Cp-V124W (p1195) showed that Cp-V124W was, similar to WT Cp, predominantly localized to the nucleus at 24 h and showed a dramatic shift to a cytoplasmic distribution (71% N < C) at 72 h post-expression (Fig. 6A, mAb3120). Unlike for WT Cp, however, the anti-HBc polyclonal antibody showed a distribution pattern similar to mAb3120 for all conditions, consistent with rapid assembly of Cp-V124W, and it being associated with capsids and/or high-order assemblages at all time points.

10.1128/mbio.03303-22.3 MOVIE S3 Representative video corresponding to Fig. 5 showing live cell imaging of WT Cp-V142W-NG expression in a Huh7 cell, with images acquired every 30 min over 67.5 h of continual imaging. Arrows and outlines highlight a cell division event. Download Movie S3, AVI file, 0.8 MB. Copyright © 2023 Romero et al. 2023 Romero et al. https://creativecommons.org/licenses/by/4.0/ This content is distributed under the terms of the Creative Commons Attribution 4.0 International license.

Live cell imaging using a fluorescently tagged Cp-V124W-NG derivative showed, similar to WT Cp-NG, rapid early accumulation in the nucleus but, unlike WT Cp-NG, an even more dramatic localization to distinct nuclear bodies that we confirmed to be nucleoli using an anti-Nucleophosmin (B23) antibody (Fig. 6C). During cell division, nucleoli-associated Cp-V124W-NG relocalized to the cytoplasm, and then the structures dissipated, followed by a gradual return of Cp-V124W-NG to the nucleus and nuclear bodies over time (Fig. 6B and Movie S3). Taken together, our analysis of the Cp-V124W rapid assembly mutant suggested that Cp’s trafficking to and accumulation within the nucleus is a fast and constitutive process and may be linked to nucleolar biology as previously proposed (24, 45–47).

## DISCUSSION

**FIG 7:**
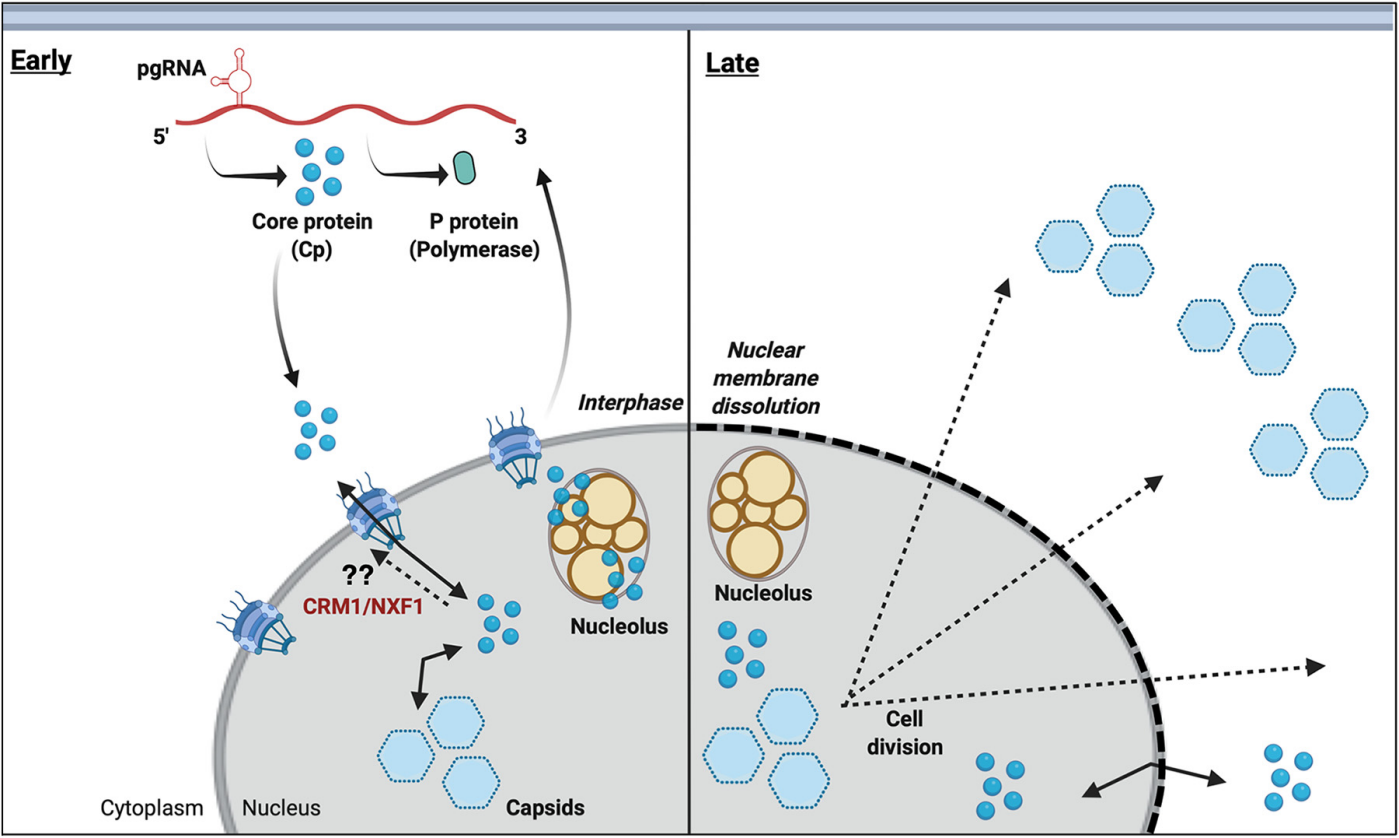
Working model for intracellular trafficking of Cp dimers and capsids in cells that are mitotically active. (Left panel) Cp monomers/dimers enter the nucleus rapidly after being translated in the cytoplasm, where they form high-order Cp assemblages (likely capsids). A significant amount of Cp is recruited to nucleoli during this phase. During interphase, Cp dimers and a subset of capsids may be capable of leaving the nucleus through the NPC using the CRM1 and/or NXF1 transport pathways. (Right panel) Provided by cell division, a large number of nuclear capsids are delivered to the cytoplasm when the nuclear envelope dissolves (dashed lines) during prometaphase, followed by cytoplasmic retention/stabilization through a yet-to-be elucidated tethering mechanism. Figure created with BioRender.com.

In this study, we applied both single dual-antibody immunofluorescence and live cell imaging to study the intracellular distribution of HBV Cp in Huh7 hepatocellular carcinoma cells, revealing that Cp distribution changes markedly over time, dependent on Cp’s ability to assemble capsids and the state of the cell cycle (see summary model in Fig. 7). Assembly-competent Cp localized to the nucleus at early time points and was shown to be able to assemble into higher-order assemblages consistent with capsids within this compartment (Fig. 2 to Fig. 4 and Fig. 7, left panel). Live cell imaging revealed that WT Cp-NG accumulated in the nucleus at early time points, and was primarily relocalized to the cytoplasm in conjunction with nuclear envelope breakdown during cell division (Fig. 4D and Fig. 7, right panel, and Movies S1 and S2). It is important to consider that cellular levels of core protein may also be a contributing factor in influencing the intracellular distribution of Cp, as demonstrated recently by Nair and Zlotnick (24) and, in this context, it is notable that we observed Cp-NG to accumulate in the nucleus even at the lowest levels of detectable expression (Fig. 4D and Movie S1). We also showed that blocking cell division with aphidicolin (APC), a drug that causes cell cycle arrest at early S phase, resulted in the entrapment of capsids/high-order assemblages in the nucleus (Fig. 5) and a rapid-assembling Cp mutant, Cp-V124W, preferentially trafficked to nucleolar compartments at early time points (Fig. 6 and Movie S3), consistent with Cp localization to the nucleus being a strong and constitutive process occurring soon after protein synthesis, followed by nuclear assembly and relocalization of capsids/high-order assemblages to the cytoplasm where they are retained after cell division.

Our conventional understanding of the HBV life cycle describes capsid assembly taking place primarily in the cytoplasm – where envelopment and viral egress also occur. Our work and that of others (19, 22, 48) now presents examples of Cp localized to the nucleus at early time points. But why? Notably, Cp harbors a nuclear localization signal (NLS) that spans the CTD (149 to 183 aa.), and is needed to direct nucleocapsids to the nucleus to initiate infection, regulated by phosphorylation (49–52). This same NLS is likely responsible for Cp’s rapid localization to the nucleus in monomer or dimer configurations soon after translation and prior to capsid assembly. Our findings also strongly suggest that the nucleus can represent a site of capsid assembly, regardless of the presence or absence of glycoproteins (Fig. 2 and 3). Importantly, a prior *in vivo* study form Guidotti, et al. also showed an early detection of Cp in the nucleus by immunohistochemical analysis of transgenic mouse hepatocytes and, consistent with our results demonstrating mitosis-dependent relocalization to the cytoplasm (19). Moreover, a study by Rabe and Kann indicated that capsids entering the nucleus disassemble and then can reassemble into capsids in the nucleus, further demonstrating that Cp subunits in the nucleus can assemble into capsids or high-order assemblages in this compartment (48). Although our study was not designed to distinguish visually between empty capsids and genome-containing nucleocapsids, we note that Yang et al. recently also detected capsids in the nucleus of HBV-expressing cells and provided evidence for nuclear capsid association with pgRNA, implicating the nucleus as a potential site for viral genome packaging (26). Future studies of nuclear Cp transport dynamics will be needed to determine if nuclear transport is obligatory to assembly or genome packaging or, alternatively, more so a consequence of the protein encoding a strong NLS.

We also observed WT Cp and Cp-V124W to localize preferentially to nucleolar compartments with unclear implications, but consistent with a subset of prior studies (24, 53). Cp-V124W’s localization to the nucleolus was particularly striking (Fig. 6), and we found it remarkable that this mutant and, likely, capsids were completely dissociated from these compartments during cell division (Fig. 6B and Movie S3). The nucleolus is the site of rRNA synthesis and processing during ribosome biogenesis (54, 55), and in theory could represent an RNA-rich environment favorable for HBV Cp binding in its capacity as an RNA-binding protein. Alternatively, the nucleolus could represent an organelle where Cp associates with host factors that promote assembly or help to coordinate pgRNA packaging. For example, Nucleophosmin (NPM1), also known as B23, a core component of the nucleolus implicated in mRNA transport, chromatin remodeling, and apoptosis, has also been shown to interact with HBV Cp and to promote capsid *in vitro*, possibly serving as an assembly priming factor (56).

If Cp’s accumulation in the nucleus (and/or in association with nucleoli) is constitutive, then how does it get out of the nucleus to reach virion egress sites in the cytoplasm? Our live cell studies reveal, for the first time, that large quantities of Cp-NG can be liberated from the nucleus during cell division and, interestingly, that these capsids/high-order assemblages end up being strongly retained in the cytoplasm, thereby preventing re-accumulation in the nucleus during anaphase (Fig. 4D). These results suggest that the cell cycle can play a more significant role in the HBV virion assembly pathway than previously appreciated, albeit with the caveat that multiple other strong prior studies have implicated the nuclear pore as a major route for Cp nuclear export (21–24). The nuclear pore complex (NPC) channel spans a radius of ~2.6 nm in diameter, and small molecules up to 60 kDa can passively diffuse across the NPC (57, 58). Despite these size limitations, larger molecules are actively exported by transport proteins including CRM1/exportin 1 (XPO1) (59, 60), and accumulating evidence indicates that HBV Cp is regulated by a nuclear export signal (NES) that potentially accesses both CRM1-dependent and -independent (e.g., NXF1-driven) export pathways (24, 49). In this context, an important observation from our study that merits follow-up is demonstration that when the cell cycle was blocked using APC, HBV capsids/high-order assemblages remained nuclear at all time points, suggesting that cell division could outweigh the NPC as the major means of Cp nuclear escape, at least under our assay conditions (Fig. 4). Consistent with these findings, a previous study indicated that cell cycle state influences whether HBV Cp is primarily found in the nucleus or cytoplasm; i.e., when the cell cycle was blocked between the G_o_/G_1_ stages nuclear accumulation of Cp was observed (39). We propose that cell division would represent an efficient means by which HBV can leave the nucleus, but emphasize that our study does not rule out potential contributions of the NPC and CRM1 or NXF1/p15-driven nuclear export pathways. Moreover, we again emphasize the important point that our imaging techniques, to date, have been unable to differentiate between genome-loaded nucleocapsids and empty capsids. Empty capsids are likely to represent the majority of cell-associated Cp (8, 9), and may traffic differently than nucleocapsids. Accordingly, visually distinguishing between these pools in both fixed and live cell imaging represents a major goal for our future research.

Overall, the relevance of the cell cycle to HBV replication and pathogenesis *in vivo* remains controversial. A study using liver tissue from patients with chronic HBV infection demonstrated a positive correlation between high Cp levels in the nucleus and high levels of viral DNA detected in patient serum (20). In efforts to elucidate the regulatory functions that core protein could have in the nucleus of infected cells, multi-omic studies reveal that the core protein interactome primarily consists of RNA-binding proteins involved in several aspects of mRNA metabolism, as well as alterations to the metabolic functions of hepatocellular carcinoma cells (61, 62). In contrast, other studies have shown a positive correlation between disease severity and high levels of HBV Cp primarily detected in the cytoplasm of hepatocytes (21, 63). HBV infection may be capable of dysregulating the cell cycle, affecting the tumorigenic environment by upregulating human oncogenes including telomerase, the Src kinase, and Neuraminidase 1 (30, 64–66). However, HBV-mediated induction of the cell cycle was, in a separate study, shown to yield reductions to viral gene expression (67) and, *in vivo*, rapidly proliferating liver cells in an HBV-infected humanized-mouse model created an antiviral environment, where cell proliferation was correlated with an overall decrease to HBV cccDNA levels (31, 68). HBV replication was also shown to be partially affected by the cell cycle in an HBV transgenic mouse model of liver regeneration, wherein partial hepatectomy was associated with reduced HBV DNA and core protein levels, but did not affect viral RNA levels (69). However, it is important to note that cytokine levels were increased in this study, so that cytokine-mediated antiviral effects are also likely to be a confounding variable *in vivo.* Taken together, further studies will be necessary to fully understand differences to the HBV replication cycle in resting and dividing hepatocytes.

## MATERIALS AND METHODS

### Molecular clones.

The HBV molecular clones used in this study (Table 1) were derived from the *ayw* HBV strain (GenBank accession no. V01460.1). WT Cp was expressed from plasmid pLJ144 that contains the complete HBV genome and derived from the parental clone pCH-9/3091 (17, 33, 70). The pLJ144 plasmid and all plasmids used in this study express the HBV pgRNA under the transcriptional control of the CMV-IE promoter. Plasmid pLJ144 does not express the envelope proteins due to two mutations: (i) the start codon of the S open reading frame (ORF) was changed from the ATG to ACG (T154C) and (ii) a premature termination codon TAA was introduced into the sixth codon of the S protein ORF (C169A) (71). Plasmid pLJ145 (Cp-minus) differs from pLJ144 due to the addition of a premature stop codon at the fourth codon of the Cp ORF, mutating GAC to TAA (16, 17). The pEL43 plasmid is a derivative of parental clone pCH-3143, and is defective for pgRNA packaging due to deletion of the upstream segment of the epsilon (Ep) sequence (72). Plasmid pEL43 is also defective in P protein expression due to introduction of a premature stop codon in the P ORF’s thirteenth codon, changing the CTG to TAG (16, 34). The pTMA153 plasmid, equivalent to pLJ144, expresses full length HBV pgRNA, Cp, P, and all 3 envelope proteins. The pTMA153 expression profile is the equivalent to TL25, with the exception of containing the Epstein-Barr virus origin of plasmid replication and the Tet operator sequence (13). Plasmid pJ305 encoded Cp-Y132A and eliminates P protein synthesis, due to a substitution to the start codon from ATG to ACG (16). Untagged version of Cp-V124W was expressed from plasmid p1195 plasmid that encoded the Cp V124W amino acid substitution in a pLJ144 backbone (17, 33, 70).

**TABLE 1:** HBV expression constructs used in this study

Plasmid	Description
pLJ144	Ep+ Cp+ P+ X+ Env−
pLJ145	Ep+ Cp− P+ X+ Env−
pEL43	Ep− Cp+ P− X+ Env−
pSR007	Ep− Cp− NG+ P− X+ Env−
pJ305	Ep− Cp(Y132A)+ P− X+ Env−
pSR009	Ep− Cp(Y132A)− NG+ P− X+ Env−
p1195	Ep+ Cp(V124W)+ P+ X+ Env−
P1199	Ep− Cp(V124W)+ P− X+ Env−
pSR012	Ep− Cp(V124W)− NG+ P− X+ Env−
pTMA153	Ep+ Cp+ P+ X+ Env+

NeonGreen (NG)-tagged versions of Cp were derived from pEL43 (WT, plasmid pSR007), pFU310 (Cp-Y132A, plasmid pSR009); or p1199 (Cp-V124W, plasmid pSR012), all expressed from a pEL43 backbone. All NG-tagged versions of Cp have been created on Ep- and P- pgRNA expressing plasmid backbones that make analyzing Cp function by trans-complementation assays possible with a “donor” pgRNA expressing plasmid that have been employed by the Loeb lab (70, 71). The NG fluorescent protein ORF was inserted between codons encoding Cp amino acids D78 and P79, flanked by glycine-rich flexible linker sequences: GGGSGGG at the NG N-terminus and GGGG at the NG C-terminus. All pSR plasmid variants were generated by PCR. All molecular clones were confirmed by sequencing across the entire restriction fragment insert.

### Cell culture and drug treatment.

The human liver carcinoma cell line Huh7 (73) was cultured in Dulbecco’s modified Eagle essential minimal medium nutrient mixture with Ham’s F-12 medium (DMEM-F12; Gibco) supplemented to a final concentration of 10% with heat-inactivated fetal bovine serum ([FBS], Sigma) and 1% penicillin-streptomycin-L-glutamine (PSG) solution (Sigma). To arrest cells at the G1/S transition, cells were incubated in DMEM-F12 supplemented with 10 μg/mL aphidicolin (APC) (Cell Signaling Technology Cat#:32774), with this APC concentration sufficient to block Huh7 cell proliferation with low cytotoxicity confirmed using the Click-it EdU assay (ThermoFisher Cat#:C10640).

### Transfections.

All transfections were performed using Lipofectamine 3000 (Invitrogen). Cells were seeded and maintained in 5% FBS DMEM/F12 supplemented with 1% PSG the day before transfection in glass-bottom 24-well plates (10 mm diameter, Cat. 30623-116. VWR), 8-well glass-bottom slides (1.0 cm^2^ growth area, IBIDI Cat. 80827), or 60-mm imaging plates (Cell Treat. Cat. 229660). On the day of transfection, cells were 60% to 80% confluent. The total mass of DNA transfected per condition was 1 μg/3.3 cm^2^ cell culture growth area. Transfection mixes were added directly to the cell culture medium in each well, with the transfection medium replaced with fresh, prewarmed complete medium (10% FBS DMEM/F12 with 1% PSG) at 4–6 h posttransfection.

### Immunofluorescence and image analysis (fixed-cell imaging).

All fixed-cell imaging experiments were carried out on cells grown in 24-well glass-bottom plates (VWR). Cells were fixed using 4% paraformaldehyde (PFA, EM Sciences), washed once in phospho-buffered saline (PBS), permeabilized using 0.2% Triton X-100, and blocked with NGB (50 mM NH_4_Cl, 2% goat serum, 2% BSA) buffer. DNA was stained with 5 ng/mL 4’,6-diamidino-2-phenylindole (DAPI; ThermoFisher) to visualize nuclei. For global Cp detection, a 1:1000 dilution of a polyclonal rabbit anti-core (anti-HBc) antiserum (Lot#:214-14, Austral Biologicals) was used prior to washing with PBS and staining with 1:1000 diluted secondary antibodies (goat anti-rabbit 488; Life Technologies). For differential Cp/capsid labeling, cells were first incubated with a 1:600 dilution of mouse anti-capsid antibody mAb3120 (Cat#: 2ZHC22, Cosmo Bio Co., LTD.) followed by secondary antibody (goat anti-mouse 647; Life Technologies) prior to incubation with the anti-HBc polyclonal and goat anti-rabbit 488 antisera. Images were captured using either 20× (NA 0.75) air or 100× (NA 1.45) oil-immersion objective lenses on a Nikon Ti-Eclipse inverted widefield epi-fluorescent deconvolution microscope (Nikon Corporation) equipped with an Orca-Flash 4.0 C11440 (Hamamatsu) camera, and with data collected using Nikon NIS Elements software (V 4.00.03). Images were processed and analyzed using FIJI/ImageJ image analysis software (74, 75), normalizing signal intensity to mock (no HBV) images to control for background. The relative subcellular distribution of Cp/capsid signal was determined for >100 cells per time point (24, 48, or 72 h), with Cp-expressing cells selected at random, and classified for nuclear (N) versus cytoplasmic (C) mean fluorescence intensity (MFI) being N > C (greater nuclear distribution), N < C (greater cytoplasmic distribution), or N = C (equal nuclear and cytoplasmic distributions).

For isolated capsid imaging, 100 μL of fraction #7 from cell lysates expressing WT Cp isolated and subjected to velocity sedimentation (see below) was plated directly onto a poly-d-lysine coated cover slip (Neuvitro Corporation; Cat#:GG-12-PDL), and left undisturbed at room temperature for 2 h. After 2 h, coverslips were washed 3 times using PBS and fixed using 4% formaldehyde at room temperature for 15 min. After fixation, coverslips were stained using mAb3120 at [1:600] and anti-HBc polyclonal at [1:1000] overnight at 4°C, then washed 3× with 1× PBS, followed by incubation with secondary antibodies: anti-mouse Alexa-fluor 647 and anti-rabbit Alexa-fluor 488 for 1 h at room temperature.

### Puncta analysis with PyImageJ and KNIME.

The puncta detection and segmentation workflow were first prototyped with the ImageJ Ops image processing framework (76) and PyImageJ (77), a Python library that wraps ImageJ’s Java code base and enables access to ImageJ from the Python environment. The workflow was later ported to the Konstanz Information Miner (KNIME) (78) to take advantage of KNIME’s intuitive workflow graphical user interface (GUI). Importantly the KNIME Image Processing extension (79) supports ImageJ and the ImageJ Ops framework. Briefly, dually labeled (mAb3120 and polyclonal antibody) are processed with a Gaussian convolution (sigma = 5), applied an Otsu threshold, objects are then labeled with connected component analysis (connection type = eight), label size filtered (min = 10 pixels, max = 2,000,000 pixels), and then measured for the desired statistics and geometry (e.g., circularity, diameter, intensity, etc.). The puncta analysis workflow is available on GitHub (https://github.com/shererlab/puncta-segmentation/).

### Live cell imaging and analysis.

All time-lapse live cell imaging experiments were performed in 8-well no.1.5H glass-bottom slides (1 cm^2^ Cat#: 80826, ibidi) at 37°C, ~50% humidity, and 5% CO_2_ housed in a Pathology Devices Live Cell stage top incubator (Pathology Devices, Inc). Cells were seeded and transfected as described above. After replacing transfection mix with fresh medium, imaging was carried out for 72 to 96 h starting at 4 h-posttransfection, with images acquired every 30 min. as previously described (80, 81). To quantify Cp-NG signals in the nuclear versus cytoplasmic compartments for single cell analyses, MFI was measured from nuclear or cytoplasmic regions of interest (ROIs) consisting of a fixed circular area (~50 μm^2^).

### Velocity sedimentation and Western blot analysis.

To validate the capacity of Cp-NG to form capsids, pSR007 (Cp-NG+, P−, X+, env−) was co-transfected with pLJ144 (Ep+, Cp+, *P*+, X+, env−) or pLJ145 (Ep+, Cp−, *P*+, X+, env−) with viral gene expression allowed to proceed for 72 h. Cells were washed in 1× PBS, and then lysed using 0.2% NP-40, 50 mM Tris-HCl, and 1 mM EDTA (pH 8.0) for 10 min at room temperature. Nuclei were isolated by centrifugation at 16,000 × *g* for 5 min at 4°C with the supernatant removed gently, representing the cytoplasmic lysate.

Cp and Cp-NG were detected by Western blot analysis from cytoplasmic lysates using monoclonal antibody 19C1-8 (Tokyo Future Style, Inc.) (82). For velocity sedimentation, 200 μL of the NP-40 lysate (out of 450 μL of total) was gently added to a 4-layer sucrose gradient (975 μL each: 15, 30, 45, 60%) prior to centrifugation at 52,000 rpm for 2.5 h in a Beckman SW60 rotor. Twelve fractions of equal volume (365 μL each) were then collected from top to bottom, and concentrated 10-fold (150 μL original fraction to 15 μL final volume) using methanol/chloroform-based protein precipitation. Briefly, 600 μL methanol was added per 150 μL fraction and mixed prior to adding 150 μL chloroform, vortexing, addition of 450 μL water, and centrifuging at >20,000 × *g* for 5 min. Subsequently, the upper aqueous layer was discarded prior to adding 650 μL methanol, inverting tubes 3 times, and centrifuging again at >20,000 × *g* for 5 min. Finally, protein pellets were dried and resuspended in 7.5 μL NP-40 lysis buffer prior to the addition of 7.5 μL 2× Laemmli sample buffer.

### Southern blot analysis.

To measure rcDNA synthesis, cells were washed with 1× PBS then lysed with 0.2% NP-40, 50 mM Tris-HCl, and 1 mM EDTA (pH 8.0) for 10 min in room temperature. The nuclei were isolated by centrifugation at 16,000 × *g* for 5 min at 4°C. Supernatants were adjusted to 2 mM CaCl_2_, and then incubated with 44 units of micrococcal nuclease to digest transfected plasmid DNA and unencapsidated HBV RNA. After 2 h, these reaction mixtures were supplemented with EDTA to 10 mM, sodium dodecyl sulfate (SDS) to 0.4%, and Pronase to 400 μg/mL. These additions terminated the micrococcal nuclease digestion and digested nucleocapsids and the P. Viral DNA was then extracted using phenol-chloroform mixture and precipitated with 17 μL 5 M NaCl, 20 μg Glycogen, and 1 mL of a 100% ethanol (83, 84) prior to electrophoresis using an 11-cm × 14-cm × 0.5-cm 1.25% agarose gel in 1× Tris-borate-EDTA buffer at 24 V for 24 h. DNA was then transferred by capillary action to a Hybond-N+ nylon membrane (Amersham Pharmacia Biotech), and cross-linked to the membrane with UV light.

For detection, membranes were incubated in Church hybridization solution (10 mM EDTA, 1% bovine serum albumin, 0.5 M NaHPO_4_, 7% SDS [pH 7.2]) for 1 h at 65°C prior to hybridization of the HBV probes. Probe set – HBV199(+) ACCGCCTCAGCTCTGTATCG; 2096(+) GACTCTAGCTACCTGGGTGGGTGT; 2446(+) GTTAGTATTCCTTGGACTCATAAGGTG; 2503(+) GTACCTGTCTTTAATCCTCATTGG – were used to detect full-length minus-strand DNA. All oligonucleotide probes were 5′ end labeled with [g-^32^P] ATP. Membrane was incubated at 55°C (dependent on oligo annealing temperature) for 4 h, washed with Church wash buffer at room temperature, and placed into phosphorimaging cassettes.
